# Inverted U-shaped relationship between coffee consumption and serum uric acid in American chronic kidney disease population

**DOI:** 10.3389/fnut.2023.1286430

**Published:** 2023-11-30

**Authors:** Jianling Song, Hong Li, Xiangdong Fang

**Affiliations:** ^1^Department of Nephrology, The Second Affiliated Hospital of Nanchang University, Nanchang, China; ^2^Department of Medical Records, The Second Affiliated Hospital of Nanchang University, Nanchang, China

**Keywords:** coffee, serum uric acid, chronic kidney disease, NHANES, U-shaped correlation

## Abstract

**Objective:**

The objective of this study was to examine the contentious relationship between coffee consumption and serum uric acid (SUA) levels, specifically within American population with chronic kidney disease (CKD).

**Methods:**

A total of 23,381 participants from the 2001–2018 National Health and Nutrition Examination Survey (NHANES) were selected for inclusion in this investigation. Linear regression models and generalized additive models with smooth curve fitting were employed to analyze the association between log coffee consumption and serum uric acid levels. Furthermore, the robustness of the findings was assessed across diverse populations.

**Results:**

The results of the linear regression analysis indicate a positive and marginally statistically significant (*p* = 0.0307) association between log coffee consumption and SUA, even after controlling for other variables. Furthermore, the generalized additive model revealed a nonlinear relationship characterized by an inverted U-shape between log coffee consumption and SUA. The inflection point, identified as 11.43 g/day, marks the point at which this relationship changes direction. Moreover, this inverted U-shaped relationship was consistently observed across various subgroups, including gender, age (<60 and ≥ 60 years), hypertensive and non-hypertensive individuals, those with and without cardiovascular disease, non-diabetic individuals, and those who consumed coffee with or without caffeine or sugar.

**Conclusion:**

An inverse U-shaped correlation has been observed between log coffee consumption and SUA levels. This finding implies that once coffee consumption surpasses a specific threshold, it promotes a decline in SUA levels.

## 1 Introduction

Coffee is widely recognized as one of the most popular beverages globally and is renowned for its high caffeine content, which has become integral to cultural traditions and social interactions (1). Apart from caffeine, coffee encompasses a multitude of biologically active phytochemicals, such as polyphenols (e.g., chlorogenic acid and lignans), fenugreek alkaloids, melanoidins formed during roasting, and trace amounts of magnesium, potassium, and vitamin B3 (niacin) (2). Recent research has provided some indications of the potential health advantages associated with coffee consumption (2). For instance, the consumption of coffee has been found to have advantageous effects on oxidative stress reduction, enhancement of intestinal flora, and regulation of glucose and fat metabolism (3–6). Furthermore, the consumption of coffee has been inversely linked to the likelihood of developing coronary heart disease, stroke, and cardiovascular-related mortality (7, 8), as well as a decreased risk of developing type 2 diabetes and tumors (9, 10).

Uric acid is the final product of purine catabolism, with serum uric acid (SUA) levels primarily influenced by purine metabolic rate and renal function (11, 12). Caffeine metabolites consist of 84% xanthine, which is ultimately converted by xanthine oxidase into uric acid (13). The current understanding of the relationship between coffee consumption and SUA remains inconclusive. Several studies have reported a negative correlation between coffee consumption and uric acid levels (14–19), while a Taiwanese study found higher SUA levels in individuals with high coffee consumption (20). Moreover, coffee consumption has been found to be unrelated to SUA levels in males and only marginally significant in females (21). Additionally, some studies have failed to observe any associations between coffee consumption and SUA levels (22, 23). Given the uncertain nature of the association between coffee consumption and SUA, further investigation is warranted to elucidate their relationship.

SUA levels exhibit an exponential correlation with the rising occurrence of chronic kidney disease (CKD) (24). Moreover, epidemiological investigations have revealed that hyperuricemia, independent of gout, can potentially contribute to the development of CKD (25, 26). Additionally, the consumption of coffee has been shown to confer renal benefits and is linked to a reduced risk of CKD (27, 28). However, the precise association between coffee consumption and serum uric acid levels in individuals with CKD remains uncertain. Therefore, this current cross-sectional study aims to elucidate the relationship between coffee consumption and SUA levels within a CKD population.

## 2 Methods

### 2.1 Study population

The National Health and Nutrition Examination Survey (NHANES) is a biennial cross-sectional survey administered by the Centers for Disease Control and Prevention (CDC) with the purpose of evaluating the health and nutritional well-being of the population in the United States. The selection of NHANES participants is carried out through a rigorous statistical methodology, involving random sampling, whereby individuals are chosen to partake in a personal structured interview within their residences, followed by a comprehensive physical examination at a mobile examination center. The NHANES protocols received approval from the Ethics Review Board of the CDC National Center for Health Statistics, and all participants in the survey provided written informed consent.

**Figure 1:**
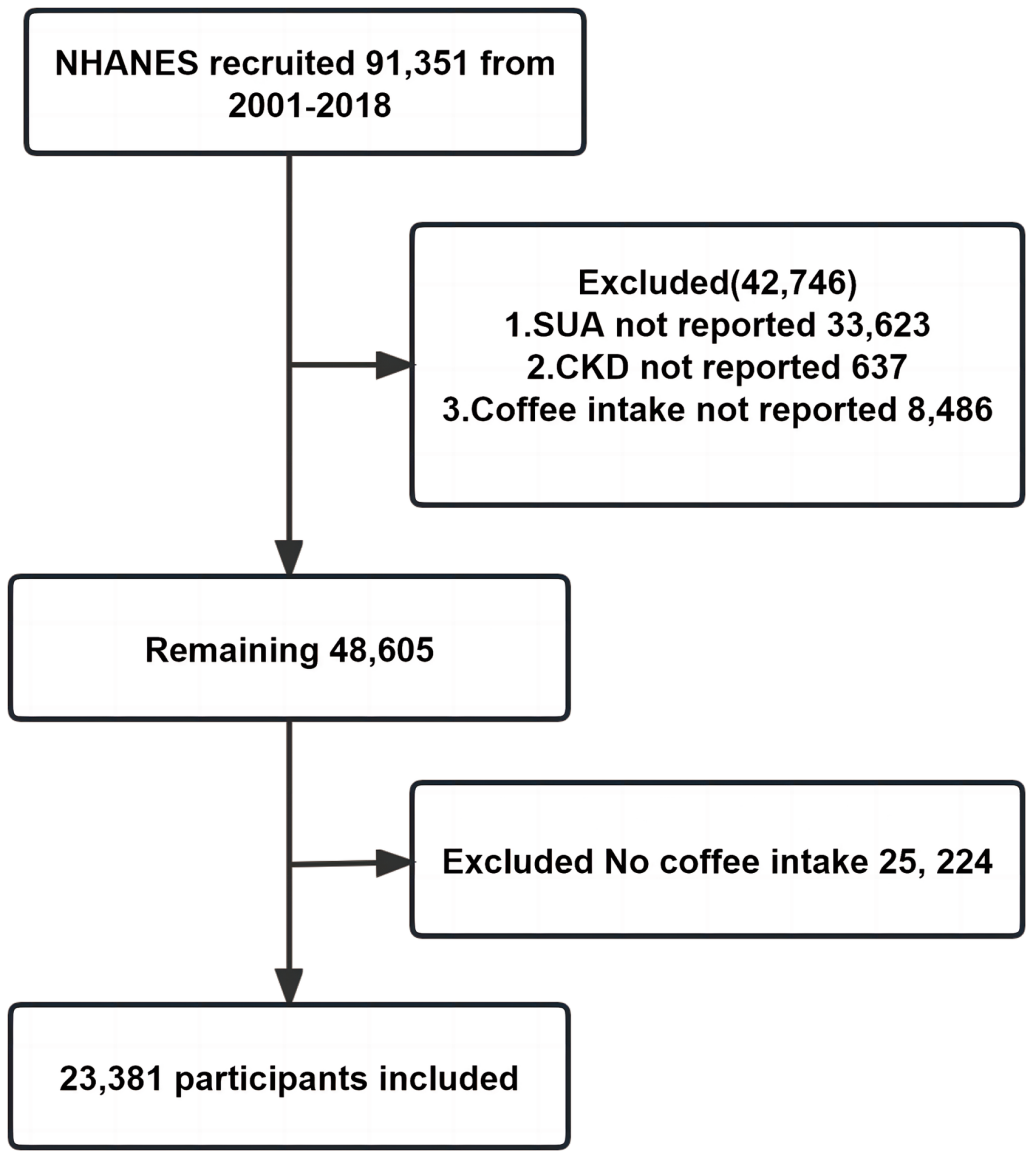
Participant inclusion flowchart.

Data from a total of 91,351 individuals spanning the years 2001 to 2018 were utilized for this study. Participants with missing values for SUA (*n* = 33,623), CKD (*n* = 637), and coffee consumption (*n* = 8,486) were excluded from the analysis. In this study, CKD was defined as estimated glomerular filtration rate (eGFR) <60 mL/min/1.73 m^2^ or urinary albumin to creatinine ratio (UACR) ≥30 mg/g (29). Additionally, individuals who did not consume coffee (*n* = 25,224) were also excluded. Notably, we retained those participants despite the presence of a missing value for one of their variables. Consequently, the final sample size for this study comprised 23,381 participants. The flowchart depicting the inclusion process undertaken by the researchers is presented in Figure 1.

### 2.2 Coffee consumption assessment

The NHANES database is utilized for the collection of data pertaining to dietary intake. Participants were requested to provide information regarding the type and quantity of food and beverages consumed within the 24-h period preceding the interview. These responses were subsequently employed to estimate the presence of 62 nutrients/food components within the aforementioned food and beverages. In the context of this particular study, the average of the 2-day food recall interviews was employed.

Food frequency questionnaires were used to determine coffee consumption in household interviews. Participants were asked “Did you drink coffee?.” Participants responded according to the following options: “none, less than 1 cup per month, 1–3 cups per month, 1 cup per week, 2–4 cups per week, 5–6 cups per week, 1 cup per day, 2–3 cups per day, 4–5 cups per day, 6 cups per day or more. 6 cups per week, 1 cup per day, 2–3 cups per day, 4–5 cups per day, 6 or more cups per day.” In the NHANES database, 1 cup = 177.6 g. Since the coffee consumption variable is non-normally distributed, we convert it to log coffee consumption. In addition, if participants answer yes to the above, then they would answer “Did you add sugar or honey?” or “Did you add artificial sweetener?” or “Was milk added to your coffee?” or “Was cream or half and half added?.” If the participant answered no, then they were assumed to not add sugar, milk, or cream to their coffee.

### 2.3 Measurement of SUA and other covariates

The NHANES database from 2001 to 2018 offers comprehensive data on various biomarkers including fasting blood glucose (FBG), hemoglobin A1C (HBA1C), SUA, total cholesterol (TC), triglyceride (TG), high-density lipoprotein (HDL), low-density lipoprotein (LDL), and creatinine (Cr). Moreover, it also provides measurements of urinary creatinine and urinary albumin, enabling the calculation of UACR. Additionally, eGFR was determined using the CKD-EPI formula (30), while body mass index (BMI) was derived from standardized measurements of weight and height.

Furthermore, the demographic data encompasses variables such as age, gender (male or female), and ethnicity, which comprises categories such as Mexican American, non-Hispanic White, non-Hispanic Black, and other (including other Hispanic and other Race-Including Multi-Racial).

Past medical history was defined from participants’ physical exams, self-reports, or based on the prescription medications they were taking. Briefly, hypertension was defined as 3 blood pressure measurements (systolic blood pressure ≥ 140 mmHg or diastolic blood pressure > 90 mmHg) or an affirmative answer to the following questions: “Ever told you had high blood pressure,” “Taking prescription for hypertension.” Diabetes was defined as an affirmative answer to the following questions: “Doctor told you have diabetes,” “Taking insulin now,” “Taking diabetic pills to lower blood sugar.” Cardiovascular disease (CVD) was defined as an affirmative answer to the following questions: “Ever told had congestive heart failure,” “Ever told you had coronary heart disease,” “Ever told you had coronary heart disease,” “Ever told you had angina/angina pectoris “. In addition, data on participants’ use of prescription medications came from their self-reports.

Drinking is now defined as responding yes to the following question: “Had at least 12 alcohol drinks/1 year?,” otherwise it is defined as not drinking. Currently smoking was defined as an affirmative answer to the following questions “smoking ≥100 lifetime cigarettes” and “currently smoking cigarettes every day or some days or smoking a cigarette in the past 5 days,” otherwise defined as non-smoking. Former smoking was defined as “smoking ≥100 lifetime cigarettes” and “currently not smoking at all and does not report smoking a cigarette in the past 5 days.” Sport level is derived from the participant’s reported level of activity intensity: mild or vigorous.

### 2.4 Statistical analysis

The initial analysis examined disparities in the characteristics of participants primarily based on their status as CKD patients. Continuous variables were represented using survey-weighted medians (Q1, Q3), while *p*-values were determined through survey-weighted linear regression (function svyglm in R language). Categorical variables were represented using survey-weighted percentages, and *p*-values were calculated using survey-weighted chi-square tests (function svytable in R language).

To evaluate the correlation between log coffee consumption and SUA levels, three weighted multiple linear regression models were employed, incorporating asymptotic adjustment levels. Each model featured log coffee consumption as the primary independent variable and SUA as the dependent variable. The crude model lacked any adjustments, while model 1 accounted for gender, age, and race. Model 2, on the other hand, considered HBA1C, TG, HDL, LDL, UACR, eGFR, BMI, caffeine, hypertension, diabetes, CVD, smoking, drinking, antihypertensive therapy, glucose-lowering therapy, urate-lowering therapy, antiplatelet aggregation therapy, diuretics as additional factors. It is noteworthy to mention that our covariate screening process involved evaluating the impact of introducing or removing covariates from the base and full models. This evaluation was based on whether the regression coefficient of log coffee consumption exceeded 10% or if the *p*-value of the regression coefficient of the covariates with the SUA was determined to be less than 0.1.

Furthermore, this study employed a generalized additivity model (GAM) and restricted cubic spline (smoothed curve fitting) to investigate the nonlinear relationship between log coffee consumption and SUA levels. In cases where a nonlinear relationship was detected through the smoothed curve fitting, a recursive algorithm was implemented to estimate the inflection point. A two-piecewise linear regression model was then constructed to determine the threshold effect. To determine whether the optimal model exhibited linearity or nonlinearity, a log-likelihood ratio test was conducted to calculate the *p* values for nonlinearity.

In order to enhance the reliability of the findings, the following analyses were conducted: (1) Covariates with missing values were assigned dummy variables and incorporated into the multiple regression model and two-piecewise linear regression model for adjustment. (2) Stratified analyses were carried out to examine various subpopulations, encompassing gender, age, and the presence of hypertension, diabetes, and CVD. (3) Stratified analyses were conducted to explore the impact of caffeine, milk, and cream content in coffee.

Statistical analyses were performed by incorporating sampling weights to accommodate the intricacies of the NHANES database survey design. The software tools employed for conducting these analyses were R (version 4.3.1) and Empower Stats (version 4.2).

## 3 Results

### 3.1 Weighted demographic characteristics of participants and baseline characteristics

A total of 23,381 participants, consisting of 4,695 individuals with CKD and 18,686 individuals without CKD, were selected from the 2001–2018 NHANES. Table 1 presents the weighted demographic characteristics of the participants and the distribution of other covariates based on the CKD subgroups. The median age, expressed as the interquartile range, was 67.00 (53.00, 77.00) years for individuals with CKD and 48.00 (36.00, 59.00) years for individuals without CKD. In the CKD group, the proportions of males and females were 42.24% and 57.76%, respectively. And, in the non-CKD group, the proportions of males and females were 48.74% and 51.26%, respectively. Non-Hispanic white had the highest percentage of the two groups. Additionally, the median SUA levels were 350.90 (291.50,416.40) μmol/L for individuals with CKD and 309.30 (255.80, 368.80) μmol/L for individuals without CKD. The median daily coffee consumption for individuals with CKD was 386.60 (237.60, 615.00) g/day, while for those without CKD it was 429.20 (245.05, 683.40) g/day. The median log coffee consumption for the CKD group was 5.96 (5.47, 6.42) g/day, compared to 6.06 (5.50, 6.53) g/day for the non-CKD group. In the CKD group, coffee consumption with sweeten, cream, milk, and caffeine accounted for 2.09, 0.17, 100, and 14.35% of total consumption, respectively. In the non-CKD group, these proportions were 2.28, 0.56, 99.96, and 7.95%, respectively. In relation to additional baseline characteristics, individuals with CKD exhibited elevated levels of FBG, HBA1c, TG, SUA, and BMI, while displaying decreased levels of TC, HDL, and LDL in comparison to those without CKD. Furthermore, CKD participants reported a lower daily coffee consumption. Moreover, CKD participants demonstrated higher prevalence rates of hypertension, diabetes, and CVD. They also exhibited higher rates of medication usage for blood pressure control, glucose management, SUA reduction, antiplatelet aggregation, and diuretic administration. Additionally, CKD participants were more inclined to be current smokers. Although non-CKD participants exhibited a higher likelihood of engaging in drinking behavior, they also demonstrated greater levels of physical activity intensity.

**Table 1:** Participant weighted characteristics based on CKD from NHANES (2001–2018).

	Non-CKD	CKD	*p*-value
***N***	18,686	4,695	
**Age (years)**^**#**^	48.00 (36.00, 59.00)	67.00 (53.00, 77.00)	<0.0001
**Sex (*n*) %**			<0.0001
**Male**	(9,058) 48.74	(2,222) 42.24	
**Female**	(9,628) 51.26	(2,473) 57.76	
**Eth (*n*) %**			<0.0001
Mexican American	(3,612) 8.08	(709) 6.42	
Non-Hispanic Black	(2,525) 6.27	(727) 7.88	
Non-Hispanic White	(9,024) 73.65	(2,642) 76.35	
Other	(3,525) 12.00	(617) 9.35	
**FBG (mmol/L)** ^**#**^	5.50 (5.11, 5.94)	5.88 (5.33, 6.94)	<0.0001
**HBA1C (%)** ^**#**^	5.40 (5.20, 5.70)	5.70 (5.40, 6.20)	<0.0001
**TC (mmol/L)** ^**#**^	5.04 (4.40, 5.77)	4.94 (4.19, 5.79)	0.0042
**TG (mmol/L)** ^**#**^	1.15 (0.81, 1.72)	1.38 (0.96, 1.99)	<0.0001
**HDL (mmol/L)** ^**#**^	1.34 (1.11, 1.66)	1.32 (1.06, 1.63)	0.0130
**LDL (mmol/L)** ^**#**^	2.97 (2.40, 3.57)	2.74 (2.15, 3.44)	<0.0001
**UACR (mg/g)** ^**#**^	5.99 (4.15, 9.29)	38.48 (11.00, 86.00)	<0.0001
**eGFR (mL/min/1.73 m**^**2**^**)**^**#**^	95.29 (82.31, 108.29)	59.57 (50.81, 91.64)	<0.0001
**SUA (μmol/L)** ^**#**^	309.30 (255.80, 368.80)	350.90 (291.50, 416.40)	<0.0001
**BMI (kg/m**^**2**^**)**^**#**^	(18,521) 27.44	(4,555) 28.63	<0.0001
**Coffee consumption (g/day)**^**#**^	429.20 (245.05, 683.40)	386.60 (237.60, 615.00)	<0.0001
Log coffee consumption (g/day) ^#^	6.06 (5.50, 6.53)	5.96 (5.47, 6.42)	0.0099
Sweeten-added (*n*) %	(530) 2.28	(106) 2.09	0.5570
Cream-added (*n*) %	(84) 0.56	(6) 0.17	0.0092
Milk-added (*n*) %	(18,676) 99.96	(4,695) 100.00	0.2595
Caffeine (*n*) %	(2,740) 15.47	(659) 14.35	0.8363
**Previous medical history**			
Hypertension (*n*) %	(7,006) 34.81	(3,454) 70.14	<0.0001
Diabetes (*n*) %	(1,806) 7.51	(1,481) 26.72	<0.0001
CVD (*n*) %	(1,511) 7.10	(1,428) 27.17	<0.0001
**Smoking (*n*) %**			<0.0001
No	(8,682) 47.86	(2,020) 43.72	
Now	(5,072) 29.37	(1,761) 39.47	
Former	(3,997) 22.77	(790) 16.81	
**Drinking (*n*) %**			<0.0001
No	(1,781) 7.29	(633) 12.40	
Now	(14,864) 92.71	(3,618) 87.60	
**Sport lever (*n*) %**			0.0095
Mild	(3,048) 87.43	(654) 91.62	
Vigorous	(534) 12.57	(74) 8.38	
**Drugs (*n*) %**			
Glucose-lowering therapy	(345) 2.27	(189) 3.61	0.0002
Antihypertensive therapy	(1,857) 15.32	(1,182) 27.80	<0.0001
Hypolipemic drug therapy	(784) 7.38	(243) 6.48	0.1368
Urate-lowering therapy	(95) 1.05	(127) 2.94	<0.0001
Antiplatelet aggregation therapy	(234) 1.72	(235) 5.30	<0.0001
Diuretic	(630) 5.41	(413) 9.90	<0.0001

Eth, ethnic; FBG, fasting blood glucose; HBA1C, glycated hemoglobin; TC, total cholesterol; TG, triglyceride; HDL, high-density lipoprotein cholesterol; LDL, low-density lipoprotein cholesterol; UACR, urinary albumin-to-creatinine ratio; eGFR, estimated glomerular filtration rate; SUA, serum uric acid; BMI, body mass index; CVD, cardiovascular disease. #:survey-weighted medians (Q1, Q3). Otherwise expressed using survey-weighted percentages.

### 3.2 Association of log coffee consumption with SUA in CKD participants

The association between coffee consumption and SUA levels in individuals with CKD was examined using multivariate linear regression analysis and two-piecewise linear regression analysis, as presented in Table 2. The results of the multivariate linear regression analysis indicated that for every 1-g/day increase in log coffee consumption, there was a corresponding increase of 8.85 (95% CI: 5.32, 12.38) μmol/L in SUA levels in the crude model, which was not adjusted for other variables. In Model I, an increase of 1-g/day in log coffee consumption was found to be associated with a 2.04 (95% CI: −1.46, 5.55) μmol/L increase in SUA. Model II revealed that a 1-g/day increase in log coffee consumption was associated with a 6.07 (95% CI: 0.57, 11.58) μmol/L increase in SUA. Furthermore, in order to account for the impact of missing variable values on the findings and prevent a reduction in sample size, dummy variables were adjusted in Model III. The results indicated that a 1-g/day increase in log coffee consumption was associated with a 1.43 (95% CI: −1.69, 4.55) μmol/L increase in SUA.

**Table 2:** Multivariate linear regression and two-piecewise linear regression to analyze the effect of log coffee consumption (g/day) on serum uric acid (μmol/L) in CKD participants.

Outcome	SUA (μmol/L)		
	*β* (95% CI)	*p*-value	*p* nonlinear value (*p* for log-likelihood ratio test)
Crude model			
Multivariate linear regression	8.85 (5.32, 12.38)	<0.0001	–
Two-piecewise linear regression			0.001
Log coffee consumption <6.89	11.93 (7.94, 15.92)	<0.0001	
Log coffee consumption >6.89	−25.68 (−46.87, −4.49)	0.0176	
Model I			
Multivariate linear regression	2.04 (−1.46, 5.55)	0.2523	–
Two-piecewise linear regression			0.005
Log coffee consumption <6.96	4.41 (0.55, 8.28)	0.0253	
Log coffee consumption >6.96	−29.51 (−51.67, −7.34)	0.0091	
Model II			
Multivariate linear regression	6.07 (0.57, 11.58)	0.0307	–
Two-piecewise linear regression			<0.001
Log coffee consumption <6.96	11.43 (5.50, 17.36)	0.0002	
Log coffee consumption >6.96	−65.77 (−97.13, −34.41)	<0.0001	
Model III			
Multivariate linear regression	1.43 (−1.69, 4.55)	0.3688	–
Two-piecewise linear regression			<0.001
Log coffee consumption <6.87	4.49 (1.01, 7.97)	0.0115	
Log coffee consumption >6.87	−31.01 (−47.87, −14.14)	0.0003	

No adjustment for any variables in the crude model. Model I adjusts for sex, age, and ethnic. Model II adjusts Model I + HBA1C, TG, HDL, LDL, UACR, eGFR, BMI, caffeine, hypertension, diabetes, CVD, smoking, drinking, antihypertensive therapy, glucose-lowering therapy, urate-lowering therapy; antiplatelet aggregation therapy; diuretics. Model III adjusts Model II + HBA1C dummy variable, TG dummy variable, HDL dummy variable, LDL dummy variable, UACR dummy variable, eGFR dummy variable, BMI dummy variable, caffeine dummy variable, hypertension dummy variable, diabetes dummy variable, CVD dummy variable, smoking dummy variable, drinking dummy variable, antihypertensive therapy dummy variable, glucose-lowering therapy dummy variable, urate-lowering therapy dummy variable; antiplatelet aggregation therapy dummy variable; diuretics dummy variable.

**Figure 2:**
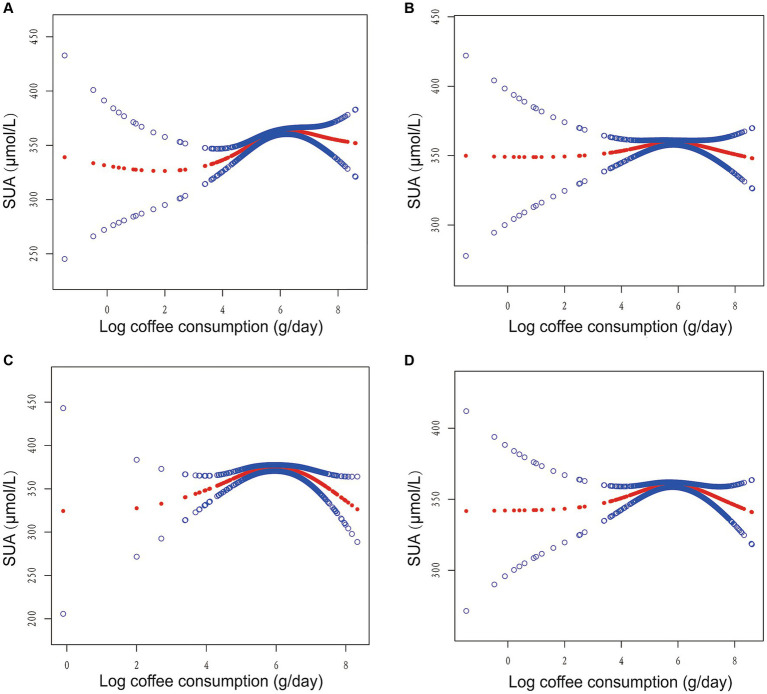
Association between log coffee consumption (g/day) and serum uric acid (µmol/L) in CKD participants. **(A)** No adjustment for any variables in the crude model. **(B)** Model I adjusts for sex, age, and ethnic. **(C)** Model II adjusts Model I + HBA1C, TG, HDL, LDL, UACR, eGFR, BMI, caffeine, hypertension, diabetes, CVD, smoking, drinking, antihypertensive therapy, glucose-lowering therapy, urate-lowering therapy; antiplatelet aggregation therapy; diuretics. **(D)** Model III adjusts Model II + HBA1C dummy variable, TG dummy variable, HDL dummy variable, LDL dummy variable, UACR dummy variable, eGFR dummy variable, BMI dummy variable, caffeine dummy variable, hypertension dummy variable, diabetes dummy variable, CVD dummy variable, smoking dummy variable, drinking dummy variable, antihypertensive therapy dummy variable, glucose-lowering therapy dummy variable, urate-lowering therapy dummy variable; antiplatelet aggregation therapy dummy variable; diuretics dummy variable.

Furthermore, we employed GAM with a smoothed curve fitting technique to estimate the non-linear association between log coffee consumption and SUA levels, as depicted in Figure 2. Intriguingly, our analysis using a two-piecewise linear regression model revealed a non-linear correlation between log coffee consumption and SUA, exhibiting an inverted U-shaped curve across all models. The identification of the inflection point was accomplished through threshold effect analysis, as presented in Table 2. Briefly, in the crude model, it was observed that individuals who consumed less than 6.89 g/day of log coffee experienced an increase in SUA by 11.93 (95% CI: 7.94, 15.92) μmol/L for every 1-g/day increment in log coffee consumption. Inversely, when log coffee consumption exceeded 6.89 g/day, a decrease in SUA by 25.68 (95% CI: −46.87, −4.49) μmol/L was observed for each 1-g/day increase in log coffee consumption. In Model I, individuals with a log coffee consumption of less than 6.96 g/day experienced a significant increase in SUA levels by 4.41 (95% CI: 0.55, 8.28) μmol/L for every 1-g/day increase in log coffee consumption. Conversely, when log Coffee consumption exceeded 6.96 g/day, there was a notable decrease in SUA levels by 29.51 (95% CI: −51.67, −7.34) μmol/L for each 1-g/day increase in log coffee consumption. In Model II, it was observed that individuals with a log coffee consumption below 6.96 g/day experienced a significant increase in SUA levels of 11.43 (95% CI: 5.50, 17.36) μmol/L for every 1-g/day increase in log coffee consumption. But, when log coffee consumption surpassed 6.96 g/day, there was a notable decrease in SUA levels of 65.77 (95% CI: −97.13, −34.41) μmol/L for each 1-g/day increase in log coffee consumption. In Model III, within the subset of individuals whose log coffee consumption was below 6.87 g/day, a statistically significant positive association was observed between each 1-g/day increase in log coffee consumption and a corresponding increase in SUA levels of 4.49 (95% CI: 1.01, 7.97) μmol/L. However, when log coffee consumption exceeded 6.87 g/day, a significant negative association was found, with each 1-g/day increase in log coffee consumption associated with a decrease in SUA levels of 31.01 (95% CI: −47.87, −14.14) μmol/L.

### 3.3 Association between log coffee consumption and SUA in different subgroups

**Figure 3:**
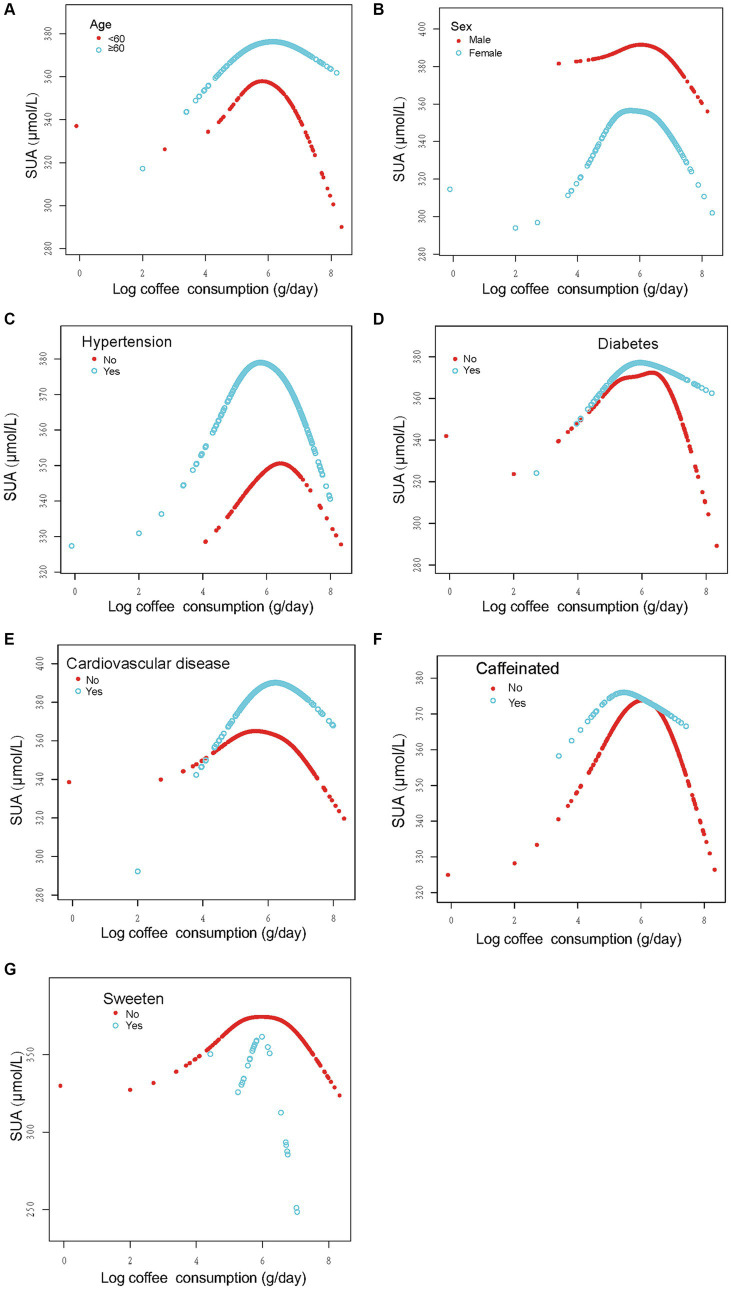
Stratified analysis of the association between log coffee consumption (g/day) and serum uric acid (μmol/L) in CKD participants. **(A–G)**: sex, age, and ethnic, HBA1C, TG, HDL, LDL, UACR, eGFR, BMI, caffeine, hypertension, diabetes, CVD, smoking, drinking, antihypertensive therapy, glucose-lowering therapy, urate-lowering therapy; antiplatelet aggregation therapy and diuretics were adjusted. For the sex, age, hypertension, diabetes, cardiovascular disease, and caffeine subgroups, the model adjusted for factors other than sex, age, hypertension, diabetes, cardiovascular disease, and caffeine, respectively.

In order to evaluate the reliability of the findings, subgroup analyses were conducted considering variables such as sex, age, past medical history (including hypertension, diabetes, and CVD), and the presence of caffeine, milk, cream, and sweeten in coffee. The results revealed a consistent inverted U-shaped association between log coffee consumption and SUA across various subgroups, including sex (<60 and ≥ 60 years), gender (male and female), non-diabetes, presence or absence of hypertension, presence or absence of CVD, and the presence or absence of caffeine or sweeten in coffee (Figure 3). Furthermore, the inflection points of the threshold effects analysis within various subgroups, as determined through measurements obtained from the two-piecewise linear regression model, are illustrated in Table 3. Additionally, an examination of the linear trends between log coffee consumption and SUA within different subgroups was conducted, and the outcomes are presented in Supplementary Table S2. However, the lack of a sufficient sample size prevented us from calculating whether there was an inverted U-shaped association between log coffee consumption and SUA in subgroups categorized by the presence or absence of milk and cream in coffee. Last, we examined the association between log coffee consumption and SUA in non-CKD participants. However, no inverted U-shaped relationship was observed in non-CKD participants (Supplementary Figure S1; Supplementary Table S3).

**Table 3:** Two-piecewise linear regression methods analyzed the association between log coffee consumption (g/day) and serum uric acid levels (μmol/L) in different subgroups in CKD participants.

Outcome	SUA (μmol/L)		
	Adjusted *β* (95% CI)	*p*-value	*p* nonlinear value (*p* for log-likelihood ratio test)
Age < 60			0.008
Log coffee consumption <6.97	5.47 (−7.03, 17.98)	0.3916	
Log coffee consumption >6.97	−66.15 (−117.53, −14.77)	0.0123	
Age ≥ 60			0.003
Log coffee consumption <6.91	15.34 (8.35, 22.33)	<0.0001	
Log coffee consumption >6.91	−44.86 (−83.34, −6.39)	<0.0001	
Male			<0.001
Log coffee consumption <7.07	11.46 (2.59, 20.34)	0.0116	
Log coffee consumption >7.07	−104.90 (−151.22, −58.58)	<0.0001	
Female			0.015
Log coffee consumption <5.26	27.60 (9.72, 45.47)	0.0026	
Log coffee consumption >5.26	−1.04 (−11.39, 9.30)	0.8432	
Non-hypertension			0.131
Log coffee consumption <6.58	9.87 (−6.40, 26.14)	0.2357	
Log coffee consumption >6.58	−22.85 (−59.23, 13.53)	0.2197	
Hypertension			<0.001
Log coffee consumption <6.96	11.63 (5.01, 18.25)	0.0006	
Log coffee consumption >6.96	−79.34 (−120.02, −38.66)	0.0001	
Non-diabetes			<0.001
Log coffee consumption <6.95	8.72 (1.55, 15.89)	0.0173	
Log coffee consumption >6.95	−86.29 (−123.60, −48.98)	<0.0001	
Diabetes			0.434
Log coffee consumption <4.79	38.07 (−22.92, 99.06)	0.2217	
Log coffee consumption >4.79	12.78 (2.10, 23.46)	0.0194	
Non-cardiovascular disease			<0.001
Log coffee consumption <6.87	8.45 (1.28, 15.62)	0.0211	
Log coffee consumption >6.87	−64.06 (−95.93, −32.20)	<0.0001	
Cardiovascular disease			0.016
Log coffee consumption <7.02	21.37 (10.73, 32.01)	<0.0001	
Log coffee consumption >7.02	−65.45 (−134.54, 3.64)	0.0639	
Caffeine-free			<0.001
Log coffee consumption <7.02	9.83 (3.52, 16.13)	0.0023	
Log coffee consumption >7.02	−76.10 (−110.31, −41.90)	<0.0001	
Caffeine			0.025
Log coffee consumption <5.22	72.85 (14.40, 131.31)	0.0155	
Log coffee consumption >5.22	−6.19 (−30.70, 18.32)	0.6211	
Sweeten-free			<0.001
Log coffee consumption <6.96	13.65 (7.76, 19.54)	<0.0001	
Log coffee consumption >6.96	−69.76 (−101.07, −38.45)	<0.0001	
Sweeten			0.004
Log coffee consumption <6.22	−42.88 (−132.45, 46.70)	0.4013	
Log coffee consumption >6.22	−162.09 (−324.45, 0.26)	0.1220	

Sex, age, and ethnic, HBA1C, TG, HDL, LDL, UACR, eGFR, BMI, caffeine, hypertension, diabetes, CVD, smoking, drinking, antihypertensive therapy, glucose-lowering therapy, urate-lowering therapy; antiplatelet aggregation therapy and diuretics were adjusted. For the sex, age, hypertension, diabetes, cardiovascular disease, and caffeine subgroups, the model adjusted for factors other than sex, age, hypertension, diabetes, cardiovascular disease, and caffeine, respectively.

## 4 Discussion

In this study, we investigated the association between coffee consumption and SUA levels in individuals with CKD. Notably, this study is the first to examine this relationship. Our findings revealed a distinctive inverted U-shaped pattern between log coffee consumption and SUA. Specifically, an increment of 1-g in log coffee consumption, when log coffee consumption was below 6.96 g/day, resulted in an elevation of SUA by 11.43 μmol/L. Conversely, when log coffee consumption exceeded 6.96 g/day, an increase of 1-g in log coffee consumption led to a reduction in SUA by 65.77 μmol/L. Furthermore, the correlation between log coffee consumption and SUA exhibited an inverted U-shaped pattern across various subgroups, including age, sex, hypertension, diabetes, CVD, and caffeine as well as sugar in coffee. Lastly, we also found inflection points in different subgroups through threshold effects analysis.

Numerous scholarly investigations have delved into the association between coffee consumption and SUA levels. Nevertheless, the outcomes derived from these studies exhibit a lack of complete consistency. Certain studies have identified an inverse correlation between coffee consumption and SUA levels (14–19), while others have observed a positive correlation (20). Additionally, a subset of studies has concluded that no discernible relationship exists between coffee consumption and SUA levels (21–23). However, it is worth noting that these studies share common methodological limitations. Firstly, none of them have investigated the potential nonlinear association between coffee consumption and SUA levels. Secondly, all of these studies have solely focused on analyzing data from the general population, rather than specifically examining individuals with CKD. This may account for the inconsistency in the scholars’ findings.

The chemical composition of coffee is intricate, encompassing caffeine, phenolic compounds, and over 90 volatile components, among others (31, 32). Caffeine, as the principal constituent of coffee, has been extensively explored in various studies investigating its association with SUA levels. However, these studies lack consensus and display heterogeneity in their findings. Bae et al. (21) observed a positive correlation between caffeine consumption and elevated SUA levels in women, while Liu et al. (33) identified a curvilinear relationship between caffeine intake and SUA, with a turning point at 60.5 mg/d. Despite the existence of preliminary research indicating that caffeine (1,3,7-trimethylxanthine) can influence SUA levels and the risk of developing gout by competitively inhibiting xanthine oxidase and exerting diuretic effects (34, 35), the intricate composition of coffee and its constituents poses challenges in discerning whether the impact of caffeine on SUA is solely attributable to caffeine itself or to other components present in coffee (including caffeine) or their interactions. In the present study, the coffees were categorized based on their caffeine content, and it was observed that the logarithmic coffee consumption exhibited a nonlinear relationship with SUA, following an inverted U-shaped pattern, irrespective of the presence or absence of caffeine in the coffee.

Furthermore, individuals exhibit a preference for enhancing the flavor of their coffee by incorporating sugar, milk, or cream. Our investigation also examined the influence of these variables on the outcomes. It was observed that the association between log coffee consumption and SUA levels persisted in a curvilinear pattern among individuals with CKD, regardless of the presence of sugar in their coffee. However, the statistical analysis of this relationship was impeded by the limited sample size of individuals who incorporated milk and cream. Therefore, the association between coffee consumption and SUA in this population needs to be explored in depth.

The consumption of coffee has been found to have an impact on chronic diseases. Notably, a moderate increase in coffee intake has been associated with a reduction in the likelihood of developing high blood pressure (36). Furthermore, individuals who consume 3 to 5 cups of coffee per day have been observed to have a significantly lower risk of CVD (8). Additionally, long-term coffee consumption has been linked to a decrease in the occurrence of type 2 diabetes (37). In a mouse model of type 2 diabetes and its corresponding control group, the consumption of coffee was found to delay weight gain and improve glucose tolerance (38). The study was stratified based on the presence or absence of hypertension, diabetes, and CVD to examine the consistency of the findings across diverse populations. The findings demonstrated a curvilinear association between log coffee intake and SUA levels in individuals with CKD, irrespective of concurrent hypertension, diabetes, or CVD.

Numerous academic studies have provided evidence supporting the advantageous effects of coffee on human health. Notably, the consumption of coffee has been linked to a decreased likelihood of developing cardiovascular disease (8). Furthermore, both caffeinated and decaffeinated coffee consumption has been found to mitigate hepatic insulin resistance caused by excessive fructose intake (6). Regarding liver health, caffeinated coffee consumption has been shown to lower hepatic collagen levels (39). Moreover, coffee has been associated with a reduced risk of various cancers, including endometrial cancer (40), hepatocellular carcinoma (41), breast cancer (42), prostate cancer (43), and melanoma (44), within the field of oncology. Furthermore, scholarly research has established a correlation between coffee consumption and a decreased likelihood of experiencing depression and suicide (45, 46). Moreover, it has been found that the intake of coffee is also linked to a lower overall mortality rate (47, 48). Consequently, existing literature indicates that incorporating coffee consumption into one’s daily routine can contribute to a healthful lifestyle (49). Specifically, the consumption of 3–5 cups of coffee per day has been shown to be advantageous in mitigating the risk of various chronic ailments (49).

To investigate the correlation between coffee consumption and SUA levels, we conducted a comprehensive analysis encompassing both linear and nonlinear approaches. In the linear regression analysis, we meticulously controlled for relevant variables to enhance the reliability of our findings. Our results indicated that an increase of 1-g/day in log coffee consumption corresponded to a 6.07 μmol/L elevation in SUA levels (*p* = 0.0307), with the statistical significance being marginally significant. Consequently, we proceeded to examine the nonlinear association in greater detail. Surprisingly, there was a significant inverted U-shaped relationship between log coffee consumption and SUA, and this relationship remained stable across populations.

Our study found that CKD participants had lower SUA levels than non-CKD participants, which is consistent with previous studies (50). It has been established that SUA plays a role in the advancement of CKD and serves as a risk factor for the disease (51, 52). Consequently, therapy aimed at reducing SUA levels may prove effective in decelerating the progression of CKD (52). Furthermore, the consumption of coffee has been found to be advantageous for individuals with CKD, as it significantly diminishes the likelihood of developing the condition (53, 54). Moderate coffee intake has been found to be correlated with a decreased likelihood of developing CKD (54). Nevertheless, the precise biological mechanism underlying this association remains uncertain. In light of the findings from our study, it is postulated that this relationship could potentially be attributed to the capacity of moderate coffee consumption to mitigate SUA levels.

In spite of certain merits, it is imperative to acknowledge the limitations of this study. Firstly, the study design employed was cross-sectional, thereby precluding the ability to establish a causal association between coffee consumption and SUA levels. To ascertain such a relationship, a protracted randomized controlled study would be necessary. Secondly, despite diligent efforts to account for factors influencing coffee consumption and SUA, certain variables remained beyond our control, including the coffee roasting technique, brewing method, and extraction duration. Additionally, a smaller proportion of study participants opted to include milk and cream in their coffee, rendering it impossible to establish a correlation between coffee consumption and SUA levels within this particular demographic. Consequently, it is imperative to conduct further investigations to elucidate the association between these variables in subsequent studies involving this population.

## 5 Conclusion

Our research has revealed a curvilinear association, specifically an inverted U-shaped nonlinearity, between log coffee consumption and SUA levels among individuals with CKD in the United States. Notably, once coffee consumption surpassed a particular threshold, SUA levels exhibited a decline.

## Data availability statement

The original contributions presented in the study are included in the article/Supplementary material, further inquiries can be directed to the corresponding author.

## Ethics statement

The NHANES protocols received approval from the Ethics Review Board of the CDC National Center for Health Statistics, and all participants in the survey provided written informed consent.

## Author contributions

JS: Investigation, Methodology, Writing – original draft. HL: Data curation, Formal analysis, Visualization, Writing – review & editing. XF: Investigation, Methodology, Resources, Writing – review & editing.
