# Supplementary material for: Inverted U-shaped relationship between coffee consumption and serum uric acid in American chronic kidney disease population
Source: Front Nutr. 2023 Nov 30;10:1286430. doi: 10.3389/fnut.2023.1286430 (PMC10719848; doi:10.3389/fnut.2023.1286430)
Supplement: Supplementary file 1 [file Data_Sheet_1.docx]

**Supplement figure 1 Association between log coffee consumption (g/day) and serum uric acid (µmol/L) in non-CKD participants**


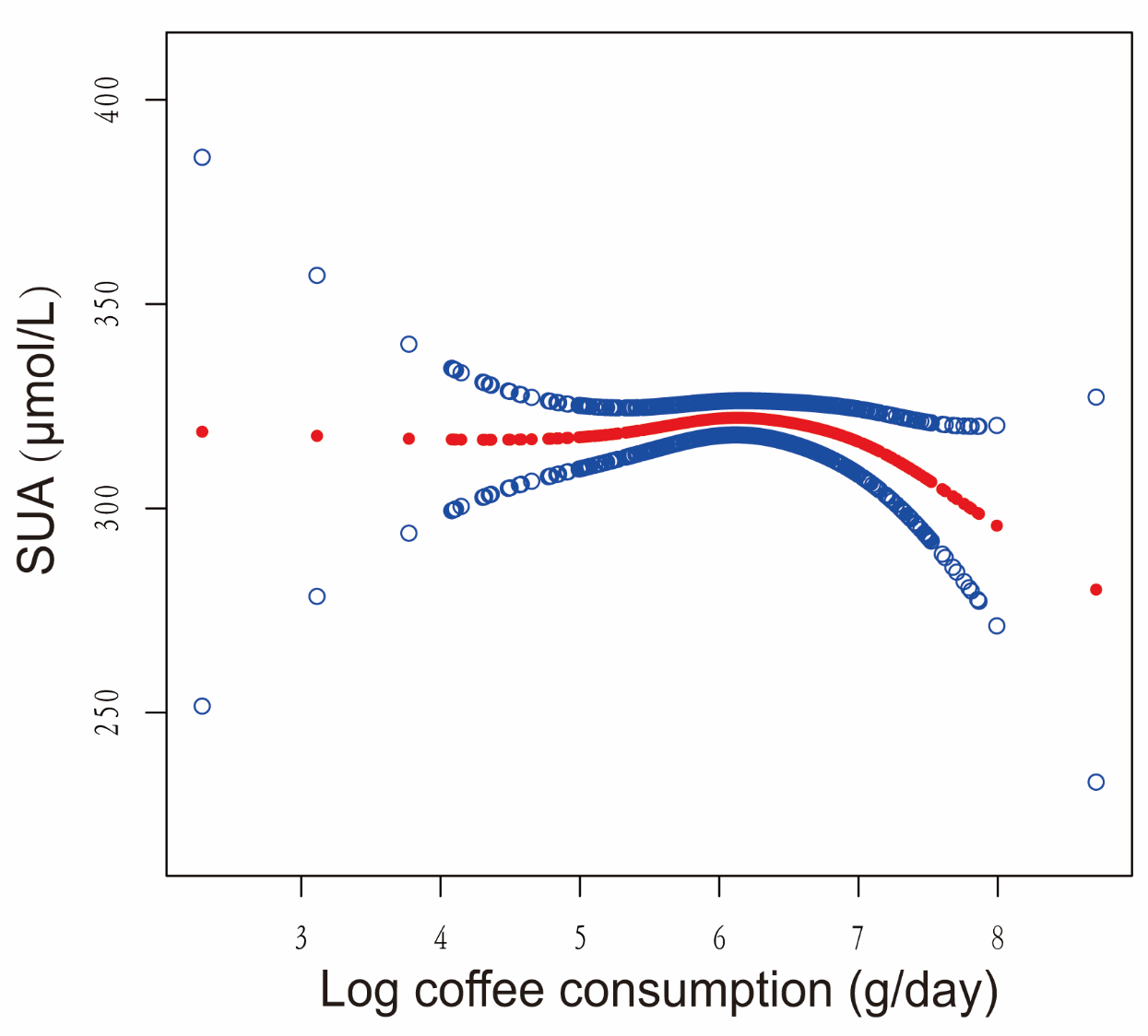


Adjusts sex, age, and ethnic, HBA1C, TG, HDL, LDL, UACR, eGFR, BMI, caffeine, hypertension, diabetes, CVD, smoking, drinking, antihypertensive therapy, glucose-lowering therapy, urate-lowering therapy; antiplatelet aggregation therapy; diuretics.
