# Supplementary material for: Inverted U-shaped relationship between coffee consumption and serum uric acid in American chronic kidney disease population
Source: Front Nutr. 2023 Nov 30;10:1286430. doi: 10.3389/fnut.2023.1286430 (PMC10719848; doi:10.3389/fnut.2023.1286430)
Supplement: Supplementary file 2 [file Data_Sheet_2.docx]

**Supplement table 1 Dummy variables for variables containing missing values**

|  | Non-CKD | CKD | P-value |
| --- | --- | --- | --- |
| FBG (mmol/L) (n) % |  |  | <0.0001 |
| <7.0 | (8,281) 45.74 | (1,647) 36.31 |  |
| ≥7.0 | (836) 3.56 | (609) 11.45 |  |
| Dummy variable | (9,569) 50.71 | (2,439) 52.24 |  |
| HBA1C (n) % |  |  | <0.0001 |
| <6.5 | (17,188) 94.33 | (3,477) 79.07 |  |
| ≥6.5 | (1,476) 5.57 | (1,212) 20.82 |  |
| Dummy variable | (22) 0.10 | (6) 0.12 |  |
| TG (mmol/L) (n) % |  |  | <0.0001 |
| <0.858 | (2,480) 14.00 | (385) 8.47 |  |
| 0.858-1.423 | (3,189) 17.25 | (798) 16.73 |  |
| >1.423 | (3,391) 17.66 | (1,062) 22.44 |  |
| Dummy variable | (9,626) 51.09 | (2,450) 52.36 |  |
| TC (mmol/L) (n) % |  |  | <0.0001 |
| <1.000 | (4,276) 21.65 | (1,406) 27.48 |  |
| 1.000-3.000 | (6,447) 34.81 | (1,491) 32.22 |  |
| >3.000 | (7,955) 43.49 | (1,797) 40.27 |  |
| Dummy variable | (8) 0.05 | (1) 0.03 |  |
| HDL (mmol/L) (n) % |  |  | <0.0001 |
| <1.16 | (5,691) 29.10 | (1,735) 34.63 |  |
| 1.16-1.47 | (6,039) 31.71 | (1,355) 28.35 |  |
| >1.47 | (6,948) 39.14 | (1,603) 36.98 |  |
| Dummy variable | (8) 0.05 | (2) 0.05 |  |
| LDL (mmol/L) (n) % |  |  | <0.0001 |
| <2.353 | (2,120) 10.81 | (716) 15.33 |  |
| 2.353-3.129 | (2,938) 16.46 | (688) 14.54 |  |
| <3.129 | (3,800) 20.62 | (764) 16.16 |  |
| Dummy variable | (9,828) 52.11 | (2,527) 53.97 |  |
| UACR (mg/g) (n) % |  |  | <0.0001 |
| <30 | (18,686) 100.00 | (1,604) 36.78 |  |
| 30-299 | - | (2,495) 52.44 |  |
| ≥300 | - | (497) 8.82 |  |
| Dummy variable | - | (99) 1.96 |  |
| eGFR (mL/min/1.73 m^2^) (n) % |  |  | <0.0001 |
| ≥90 | (11,579) 60.63 | (1,147) 26.54 |  |
| 60-89 | (7,107) 39.37 | (1,069) 21.22 |  |
| 45-59 | - | (1,625) 36.63 |  |
| 30-44 | - | (601) 11.63 |  |
| 15-29 | - | (184) 3.00 |  |
| <15 | - | (68) 0.96 |  |
| Dummy variable | (0) 0.00 | (1) 0.02 |  |
| BMI (Kg/m^2^) (n) % |  |  | <0.0001 |
| <25 | (5,640) 31.19 | (1,180) 25.58 |  |
| ≥25 | (12,881) 68.15 | (3,375) 72.26 |  |
| Dummy variable | (165) 0.66 | (140) 2.16 |  |
| Hypertension (n) % |  |  | <0.0001 |
| No | (11,670) 65.17 | (1,239) 29.84 |  |
| Yes | (7,006) 34.80 | (3,454) 70.09 |  |
| Dummy variable | (10) 0.04 | (2) 0.07 |  |
| Diabetes (n) % |  |  | <0.0001 |
| No | (16,619) 91.83 | (3,204) 73.12 |  |
| Yes | (1,806) 7.46 | (1,481) 26.66 |  |
| Dummy variable | (261) 0.71 | (10) 0.21 |  |
| CVD (n) % |  |  | <0.0001 |
| No | (16,145) 90.38 | (3,133) 71.33 |  |
| Yes | (1,511) 6.90 | (1,428) 26.61 |  |
| Dummy variable | (1,030) 2.72 | (134) 2.06 |  |
| Smoking (n) % |  |  | <0.0001 |
| No | (8,682) 46.72 | (2,020) 42.89 |  |
| Now | (5,072) 28.67 | (1,761) 38.72 |  |
| Former | (3,997) 22.22 | (790) 16.49 |  |
| Dummy variable | (935) 2.38 | (124) 1.91 |  |
| Drinking (n) % |  |  | <0.0001 |
| No | (1,781) 6.71 | (633) 11.32 |  |
| Now | (14,864) 85.36 | (3,618) 80.01 |  |
| Dummy variable | (2,041) 7.93 | (444) 8.67 |  |
| Sport lever (n) % |  |  | <0.0001 |
| Mild | (3,048) 18.65 | (654) 14.76 |  |
| Vigorous | (534) 2.68 | (74) 1.35 |  |
| Dummy variable | (15,104) 78.67 | (3,967) 83.89 |  |
| Hypolipemic drug therapy (n) % |  |  | <0.0001 |
| Yes | (784) 4.30 | (243) 5.38 |  |
| Other | (9,668) 53.94 | (3,686) 77.63 |  |
| Dummy variable | (8,234) 41.76 | (766) 16.99 |  |
| Antihypertensive therapy (n) % |  |  | <0.0001 |
| Yes | (1,857) 8.92 | (1,182) 23.08 |  |
| Other | (8,595) 49.32 | (2,747) 59.93 |  |
| Dummy variable | (8,234) 41.76 | (766) 16.99 |  |
| Glucose-lowering therapy (n) % |  |  | <0.0001 |
| Yes | (345) 1.32 | (189) 2.99 |  |
| Other | (10,107) 56.92 | (3,740) 80.02 |  |
| Dummy variable | (8,234) 41.76 | (766) 16.99 |  |
| Urate-lowering therapy (n) % |  |  | <0.0001 |
| Yes | (95) 0.61 | (127) 2.44 |  |
| Other | (10,361) 57.65 | (3,807) 80.70 |  |
| Dummy variable | (8,230) 41.74 | (761) 16.86 |  |
| Antiplatelet aggregation therapy (n) % |  |  | <0.0001 |
| Yes | (234) 1.00 | (235) 4.41 |  |
| Other | (10,222) 57.25 | (3,699) 78.74 |  |
| Dummy variable | (8,230) 41.74 | (761) 16.86 |  |
| Diuretic (n) % |  |  | <0.0001 |
| Yes | (630) 3.15 | (413) 8.24 |  |
| Other | (9,826) 55.11 | (3,521) 74.91 |  |
| Dummy variable | (8,230) 41.74 | (761) 16.86 |  |
| Caffeine (mg/day) (n) % |  |  | 0.0186 |
| ≤0 | (14,504) 76.58 | (3,754) 78.98 |  |
| >0 | (2,740) 15.47 | (659) 14.35 |  |
| Dummy variable | (1,442) 7.95 | (282) 6.67 |  |

Eth: ethnic, FBG: fasting blood glucose, HBA1C: glycated hemoglobin, TC: total cholesterol, TG: triglyceride, HDL: high-density lipoprotein cholesterol, LDL: low-density lipoprotein cholesterol, UACR: urinary albumin-to-creatinine ratio, eGFR: estimated glomerular filtration rate, SUA: serum uric acid, BMI: body mass index, CVD: cardiovascular disease.

**Supplement table 2 Multivariate linear regression analysis the association between log coffee consumption and SUA in different subgroups.**

|  | β (95%CI) | P-value | P-interaction |
| --- | --- | --- | --- |
| Sex |  |  | 0.962 |
| Male | 2.86 (-5.35, 11.07) | 0.4948 |  |
| Female | 7.72 (0.23, 15.21) | 0.0438 |  |
| Total | 6.07 (0.57, 11.58) | 0.0307 |  |
| Age |  |  | 0.034 |
| <60 | -1.47 (-12.89, 9.94) | 0.8004 |  |
| ≥60 | 11.33 (4.87, 17.79) | 0.0006 |  |
| Total | 6.94 (1.41, 12.46) | 0.0140 |  |
| Hypertension |  |  | 0.573 |
| No | 2.14 (-10.24, 14.52) | 0.7353 |  |
| Yes | 6.29 (0.12, 12.46) | 0.0458 |  |
| Total | 6.07 (0.57, 11.58) | 0.0307 |  |
| Diabetes |  |  | 0.150 |
| No | 2.26 (-4.46, 8.98) | 0.5101 |  |
| Yes | 14.60 (5.01, 24.20) | 0.0030 |  |
| Total | 6.06 (0.56, 11.56) | 0.0310 |  |
| Cardiovascular disease |  |  | 0.021 |
| No | 2.12 (-4.45, 8.70) | 0.5271 |  |
| Yes | 16.62 (6.70, 26.54) | 0.0011 |  |
| Total | 6.06 (0.56, 11.56) | 0.0310 |  |
| Caffeine |  |  | 0.152 |
| No | 4.11 (-1.77, 9.98) | 0.1708 |  |
| Yes | 12.67 (-4.64, 29.97) | 0.1530 |  |
| Total | 6.06 (0.56, 11.56) | 0.0310 |  |
| Sweeten |  |  | 0.016 |
| No | 7.78 (2.33, 13.22) | 0.0052 |  |
| Yes | -77.25 (-145.48, -9.01) | 0.0772 |  |
| Total | 7.14 (1.74, 12.53) | 0.0096 |  |

sex, age, and ethnic, HBA1C, TG, HDL, LDL, UACR, eGFR, BMI, caffeine, hypertension, diabetes, CVD, smoking, drinking, antihypertensive therapy, glucose-lowering therapy, urate-lowering therapy; antiplatelet aggregation therapy and diuretics were adjusted. For the sex, age, hypertension, diabetes, cardiovascular disease, and caffeine subgroups, the model adjusted for factors other than sex, age, hypertension, diabetes, cardiovascular disease, and caffeine, respectively.

**Supplement table 3 Multivariate linear regression and two-piecewise linear regression to analyze the effect of log coffee consumption (g/day) on serum uric acid (μmol/L) in non-CKD participants.**

| Outcome: | SUA (μmol/L) | | |
| --- | --- | --- | --- |
|  | **β (95% CI)** | **P-value** | **P nonlinear value (P for log-likelihood ratio test)** |
| Multivariate linear regression | -4.81 (-11.04, 1.42) | 0.1304 | - |
| Two-piecewise linear regression |  |  | 0.002 |
| Log coffee consumption < 6.89 | -0.95 (-7.63, 5.73) | 0.7799 |  |
| Log coffee consumption > 6.89 | -92.48 (-149.75, -35.21) | 0.0016 |  |

Adjusts sex, age, and ethnic, HBA1C, TG, HDL, LDL, UACR, eGFR, BMI, caffeine, hypertension, diabetes, CVD, smoking, drinking, antihypertensive therapy, glucose-lowering therapy, urate-lowering therapy; antiplatelet aggregation therapy; diuretics.
